# Proanthocyanidin biosynthesis and postharvest seed coat darkening in pinto bean

**DOI:** 10.1007/s11101-023-09895-8

**Published:** 2023-10-12

**Authors:** Nishat S. Islam, Sangeeta Dhaubhadel

**Affiliations:** 1 London Research and Development Centre, Agriculture and Agri-Food Canada, London, ON Canada; 2 Department of Biology, University of Western Ontario, London, ON Canada

**Keywords:** Proanthocyanidins, Postharvest seed coat darkening, Phenylpropanoid pathway, Pinto beans, Gene regulation

## Abstract

Proanthocyanidins (PAs) are polyphenolic compounds present widely in the plant kingdom. These specialized metabolites are derived from the phenylpropanoid pathway and are known for producing brown pigments in different plant organs. PAs accumulate in the seed coat tissues of flowering plants and play a determinant role in seed germination and viability, protect seeds from biotic and abiotic stresses, and thus ensure the long-term storage potential of seeds. In addition, PAs are a rich source of antioxidants for the health of both human and livestock. Many of the commercially relevant dry beans (*Phaseolus vulgaris*) contain high levels of PAs, which when oxidized cause the beans to overdarken, a phenomenon known as postharvest darkening. These darker beans give the impression of oldness, and consumers tend to avoid buying them which, in turn, affects their market value. Pinto beans, one of the leading market classes of dry beans, are affected the most by the postharvest darkening. Therefore, exploring the regulation of PA biosynthesis and accumulation in bean seed coat tissues will help to develop strategy to manage the postharvest darkening effect in pintos. In this review, we discuss the PA biosynthesis and its regulation, connecting it to seed coat color genetics for a better understanding of the mechanism of seed coat darkening.

## Introduction

Proanthocyanidins (PAs), also known as condensed tannins, are the polymers of flavan-3-ols present in a wide variety of plants, including legumes, teas, and oilseed crops. They are most abundant in the seed coats, skin of fruits, and bark of trees and are easily noticeable by their brown color when oxidized or astringent taste (Gu et al. 2004). Several studies have reported that the dietary intake of PAs confers significant health benefits to human (Shi et al. 2003; Rauf et al. 2019). PAs act as scavengers of reactive oxygen species (ROS) and thus serve as antioxidants. One of the earlier studies have found that epicatechin polyphenols (PAs) are the strongest groups of antioxidants available in plant extract and their antioxidant activity increases with the number of hydroxyl groups present in the polymer (Vinson et al. 1995). They have been shown to contain anti-carcinogenic, anti-microbial, and anti-inflammatory properties as well as help to improve flexibility in joints and provide protection from cardiovascular diseases (Duthie et al. 2000). PA supplements derived from several legumes, tea and grape seed extracts are commercially available and frequently recommended for the elderly for numerous health benefits (Rauf et al. 2019). On the other hand, higher levels of PAs lead to decreased digestion and nutrient absorption (Reddy et al. 1985). Consumption of foods with higher PAs have also been reported to inhibit iron absorption, causing anemia in the dark bean-eating population (Tako et al. 2014). Additionally, higher PA levels can impair digestibility in ruminant animals by decreasing the excretion of nitrogen to the environment and increasing protein absorption (Reed 1995; Aerts et al. 1999; Dixon et al. 2005). PAs are present exclusively in the higher divisions of the plant kingdom and deposited in selective organs, such as leaves, flowers, and seeds. Forage crops like alfalfa accumulate PAs in the vacuoles of the leaf epidermis and trichomes (Dixon and Sarnala 2020). In dry beans, PAs are present in the seed coat tissues to protect the embryo against pathogen attack, nutrient and water loss, and UV-light mediated damage (Ferreyra et al. 2012). Despite the fact that PAs have beneficial roles, they reduce seed germination efficiency (Junk-Knievel et al. 2007; Shah et al. 2018).

Pinto bean is one of the most produced and consumed beans among more than 60 different market classes in the Americas. It belongs to the Middle American landrace with a medium seed size and predominantly grown in North Dakota, Michigan and Minnesota in USA, and in Canadian prairies. Their high dietary value and affordability have made pinto beans a popular choice among other legumes. A one-half cup serving of pinto bean contains 20–30% protein and 55–65% complex carbohydrates providing around 122 kcal of energy (Suárez-Martínez et al. 2016). It serves as a source of valuable micronutrients (folate, zinc, iron, calcium, etc.) and a preferred meat substitute for vegetarians (Suárez-Martínez et al. 2016). Pinto beans (and other colored beans) are rich in flavonoids, another important class of phytonutrients (Rebello et al. 2014). Among various types of flavonoids, PAs are abundantly found in the seed coat of pinto beans.

**Fig. 1:**
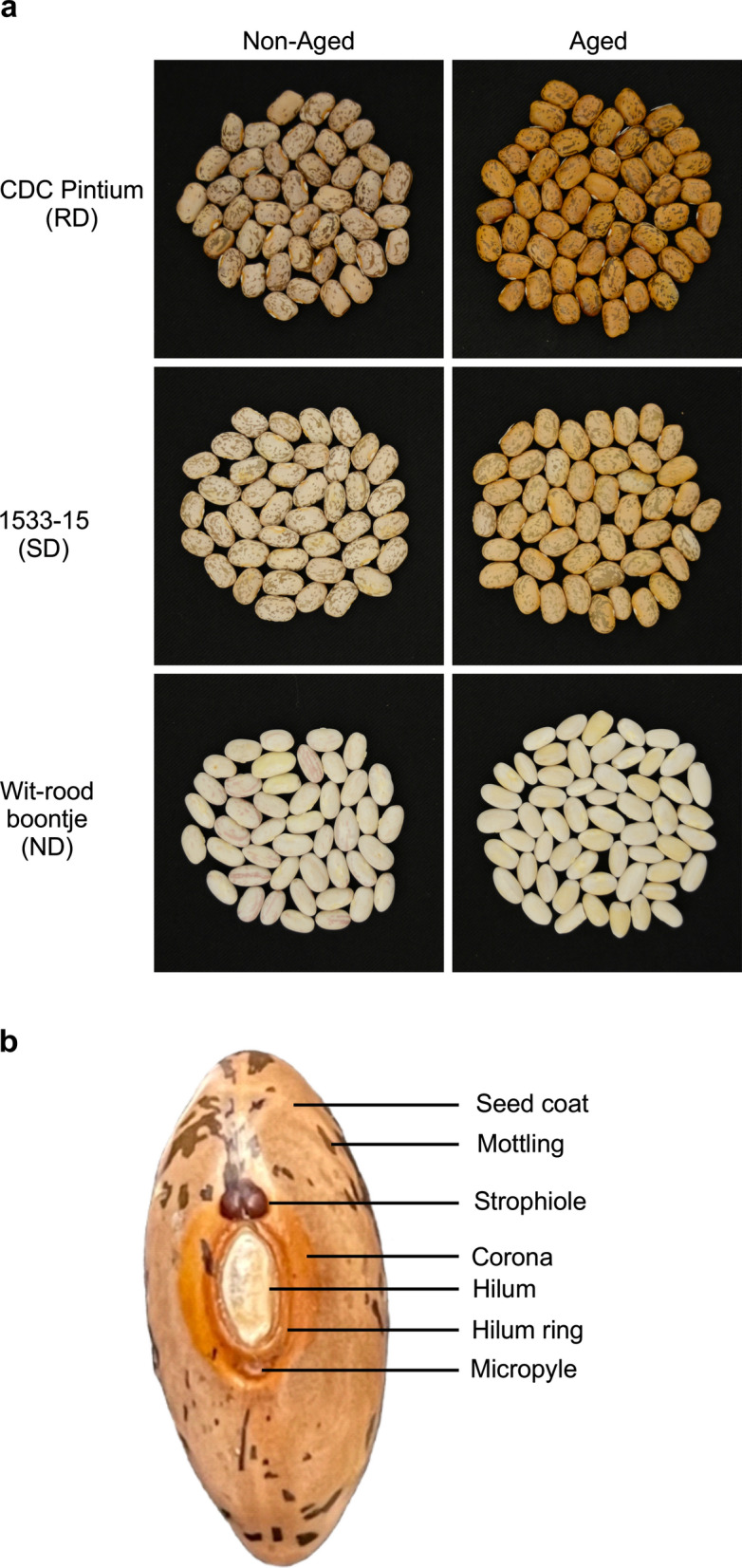
Seed coat colors in pinto beans. **a** Postharvest seed coat darkening in pinto beans. Non aged (freshly harvested) and aged (after 6 months of storage at room temperature) pictures of three different pinto cultivars: CDC Pintium (RD), 1533-15 (SD) and cranberry like Wit-rood boontje (ND). (adapted from Islam et al. 2020). **b** Area specific PA-level variation in a seed of pinto bean cultivar CDC Pintium

One of the major impacts of PAs is the postharvest seed coat darkening of several market classes of dry beans, including pintos (Beninger et al. 2005; Bassett 2007). Due to harvest delays or long-term storage, the seeds of mature beans become darker than their original creamy white color with red speckles (Park and Maga 1999). Darker beans take longer cooking and pre-cook soaking time (Miklas et al. 2020; Wiesinger et al. 2021). They also give a perception of aging, and consumers tend to avoid darker beans. Consequently, darker pinto beans are sold at a discounted price that leads to significant crop value loss to bean producers and vendors (Junk-Knievel et al. 2008; Marles et al. 2008). Based on the postharvest darkening phenotype, pinto beans (and other affected beans) are classified into three categories: regular darkening (RD), slow darkening (SD) and non-darkening (ND) (Fig. 1a). Postharvest seed coat darkening is most studied in pinto bean cultivars CDC pintium (RD), 1533-15 [also known as CDC White Mountain (SD)] and in cranberry like bean, Wit-rood boontje (ND). Multiple lines of evidence over the last two decades have attributed PA levels to postharvest darkening in pinto and pinto-like beans (Beninger et al. 2005; Alvares et al. 2019). Although postharvest seed coat darkening has been a major issue in pinto beans, this trait has been found in seeds of many crops. For example- seed coats of some cultivars of fava bean (*Vicia faba*) (Crofts et al. 1980), most market classes of lentils (*Lens culinaris*) (Dueñas et al. 2002), Kabuli chickpeas (*Cicer arietinum*), and cowpeas (*Vigna unguiculata*) (Reyes et al. 2000; Aveling and Powell 2005) change color upon aging. Cereal crops like rice, barley, and wheat seeds turn yellow to brownish upon storage which also affects their market value (Chrastil 1990; Edney et al. 1998). On the contrary, darkening is a desirable trait in some fruits and beverages and is favored by consumers. For coffee, prunes, raisins, wine, cocoa, and tea browning adds flavor (Whitaker and Lee 1955). In this review we focus on the role of PAs in postharvest seed coat darkening in pinto bean.

### Seed physiology and PA deposition in seed coat tissues

Seed dynamics play a critical role in a plant’s survival and adaptation to different environmental conditions, as well as in its overall life process and evolution. Angiosperms reproduce by a double fertilization event, and the fertilized ovule (seed) carries three genetically distinct components: the embryo, the endosperm, and the seed coat. Among the two male gametes of each pollen, one fertilizes a single ovule to form a diploid embryo (2n), and the other fuses with two polar nuclei, forming the endosperm (3n) (Coelho and Benedito 2008). The seed coat, or testa (2n), arises from two ovule integuments and the chalaza thus carries only maternal genetic material (Debeaujon et al. 2003; North et al. 2010; Baud et al. 2023). The integuments usually differentiate into three inner and two outer layers by ovular fertilization to develop as a mature seed coat which accumulates specific compounds (e.g., mucilage, flavonols, and PAs) that ensure protection to the embryo and endosperm (Baud et al. 2023).

The presence of PAs in the seed coat tissues can be determined using *p*-dimethylaminocinnamaldehyde (DMACA) and vanillin stains (Li et al. 1996; Debeaujon et al. 2003). DMACA and vanillin are two different aldehydes that react with m-diphenols of the PA monomers (catechin and epicatechins) to form a colored carbonium ion in acid (Li et al. 1996). Its deposition in different cellular layers is well studied in Arabidopsis (*Arabidopsis thaliana*), where the accumulation of PA polymers starts at the chalaza or micropyle and then spreads over the endothelial layers (Debeaujon et al. 2003; Lepiniec et al. 2006; Baud et al. 2023). In pea seed coat, PAs are deposited in the epidermal and ground parenchyma layers during seed development (Ferraro et al. 2014). In pinto beans, PAs are deposited in the epidermal or palisade layer of cells (Marles et al. 2008). Although several studies have identified differences in PA levels in CDC Pintium (RD) and 1533-15 (SD), no significant difference was observed in cell layers by histochemical or anatomical analysis (Marles et al. 2008). In pinto beans, PAs are visible at distinct locations on the seed coat: the base of the seed coat, which gives the background color; the unique mottling on the seed coat that is the identifier for the pintos; the hilum ring; the corona; the micropyle and the strophiole (Fig. 1b). All these areas vary in shades of brown, indicate different levels of PA deposition and subsequent oxidation events. However, the seed coat background and hilum color are reported to affect the canning, and storage quality of dry beans.

### Biosynthesis of PA precursors and monomers

PAs are one of the end products of the phenylpropanoid pathway. The PA biosynthesis is extensively studied in many plant species, such as *Medicago truncatula* (Dixon et al. 2005; Pang et al. 2007, 2008), *Vitis vinifera* (Adams 2006), *Lotus japonicus* (Yoshida et al. 2008), *Camellia sinensis* (Pang et al. 2013), *Glycine max* (Kovinich et al. 2012) and Arabidopsis (Winkel-Shirley 2001). Flavan-3-ols monomeric units and their linkages form the oligomers (procyanidin) or polymers of PAs. Both the type and organization of flavan-3-ols in PA polymers vary among plants (Dixon et al. 2005; Lepiniec et al. 2006). However, the most common types of procyanidins are 2,3-*cis*-(–)-epicatechin and 2,3-*trans*-(+)-catechin (Xie and Dixon 2005; Rue et al. 2018). Based on the stereochemistry and linkage positions, there are two types of oligomers: procyanidins-A type (PA-A) and the most abundant B-type (PA-B), which further polymerize to form PAs.

**Fig. 2:**
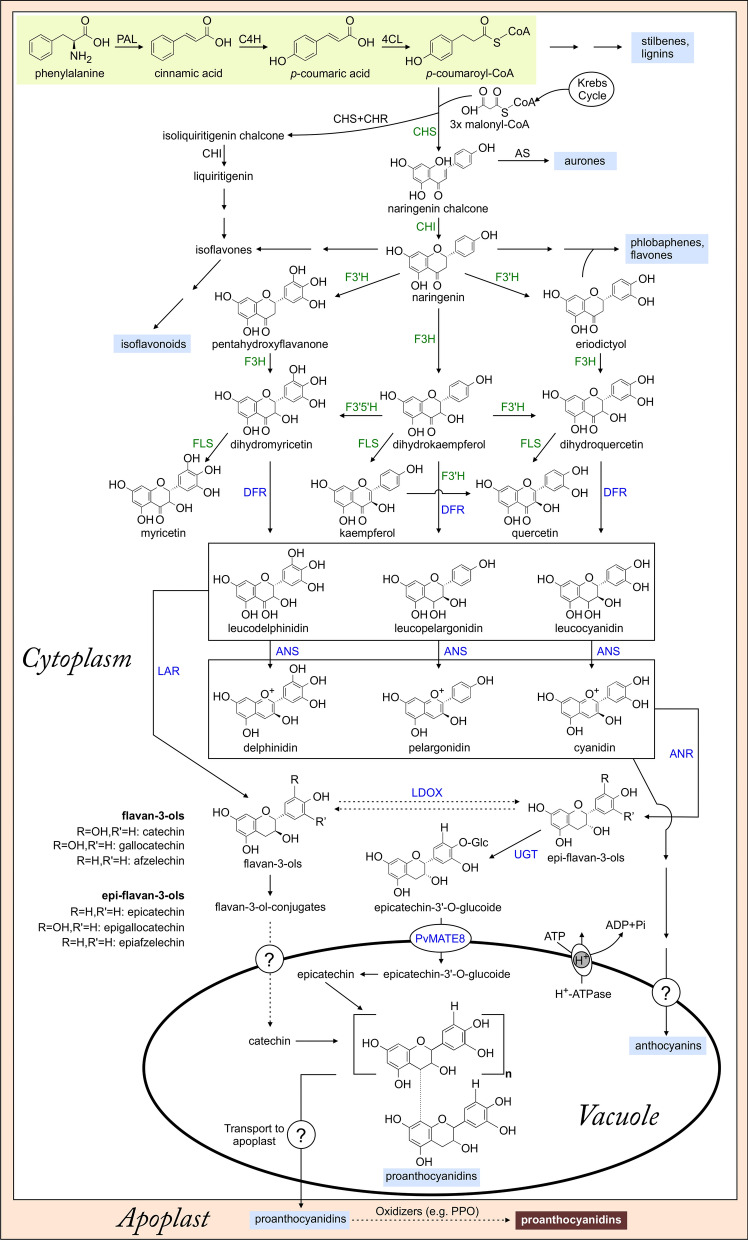
Proanthocyanidin biosynthesis in pinto bean. The dashed arrows represent speculative steps and multiple arrows indicate multiple steps. General phenylpropanoid pathway is highlighted in yellow and the end products are highlighted in blue, anthocyanin in purple and oxidized PAs in brown. Enzymes encoded by early biosynthetic genes and late biosynthetic genes are indicated by green and blue by fonts, respectively. PAL, phenylalanine ammonia lyase; C4H, cinnamate4-hydroxylase; 4Cl, 4-coumarate-CoA ligase; CHS, chalcone synthase; AS, aurone synthase; CHI, chalcone isomerase; F3′H, flavonoid 3′-hydroxylase; F3H, flavanone 3-hydroxylase; FLS, flavonol synthase; F3′5′H, flavonoid 3′,5′-hydroxylase; DFR, dihydroflavonol reductase; LAR, leucoanthocyanidin reductase; ANS, anthocyanidin synthase; ANR, anthocyanidin reductase; LDOX, leucoanthocyanidin dioxygenase; UGT, uracil glucosyltransferase; PvMATE8, multidrug and toxic compound extrusion protein 8; PPO, polyphenol oxidase

A hypothetical PA biosynthetic pathway in common bean is shown in Fig. 2. Phenylalanine derived from the shikimate pathway acts as an initial precursor molecule for the biosynthesis of all phenylpropanoids including PAs (Freixas Coutin et al. 2017; Duwadi et al. 2018). Phenylalanine ammonia-lyase (PAL) catalyzes the reaction that produces cinnamic acid which is then hydrolyzed to *p*-coumaric acid by a cinnamate 4-hydroxylase (C4H). *p*-Coumaric acid is converted to *p*-coumaroyl-coenzyme A (CoA) by 4-coumarate-CoA ligase (4CL). This three-step reaction for the biosynthesis of *p*-coumaroyl-CoA from phenylalanine is known as general phenylpropanoid pathway that provides a precursor for the synthesis of a plethora of specialized metabolites, including flavonoids i.e. anthocyanins, PA, flavonols and isoflavonoids (Ferreyra et al. 2012). In the presence of three molecules of malonyl-CoA, chalcone synthase (CHS) condenses *p*-coumaroyl-CoA to naringenin chalcone or in conjunction with chalcone reductase (CHR) produces isoliquiritigenin chalcone. Naringenin chalcone is further converted by chalcone isomerase (CHI) to naringenin, which serves as the precursor for the synthesis of PAs, isoflavonoids, phlobaphenes, and flavones. The expression levels of these early biosynthetic genes were upregulated in darker cranberry beans at early and intermediate stages of seed development leading to higher levels of PAs compared to non-darkening beans (Freixas Coutin et al. 2017).

Naringenin is hydroxylated to pentahydroxyflavanone and eriodictyol by flavonoid 3′-hydroxylase (F3′H). Flavonoid 3-hydroxylase (F3H) converts naringenin to dihydrokaempferol. Meanwhile, F3H also converts pentahydroxyflavanone to dihydromyrcetin and, eriodictyol to dihyroquercetin. Furthermore, dihydromyrcetin and dihydroquercetin can also be derived from dihydrokaempferol by the catalysis of flavonoid 3′5′-hydroxylase (F3′5′H) and F3′H, respectively. *F3′5′H* is the *V* gene in common bean that differentially affects flower and seed color (McClean et al. 2022). Flavonol synthase (FLS) catalyzes the formation of flavonols (myricetin, kaempferol and quericetin) from respective dihydroflavonols (dihydromyrcetin, dihydrokaempferol and dihyroquercetin). Among these three flavonols indicated in Fig. 2, kaempferol is found at a significantly higher level in the seed coat of CDC Pintium compared to 1533-15 (Beninger et al. 2005). Kaempferol can be hydroxylated by F3′H to produce quercetin as well.

Dihydroflavonol 4-reductase (DFR) is the branchpoint enzyme for PA and anthocyanin biosynthesis. It catalyzes the reduction of dihydroflavonols to leucoanthocyanidins/ flavan-3, 4,-diols (leucodelphinidin, leucopelargonidin and leucocyanidin). Unlike black beans, no anthocyanin is present in pinto bean seed coats (Marles et al. 2008) indicating the developmental signals to direct DFR function towards PA biosynthesis in seed coat tissues. Leucoanthocyanidins such as leucodelphinidin, leucopelargonidin and leucocyanidin are further converted to delphinidin, pelargonidin and cyanidin, respectively with the help of anthocyanidin synthase (ANS). The same leucoanthocyanidins also convert to their respective flavan-3-ols (e.g. catechin) with the help of leucoanthocyanidin reductase (LAR). The products of ANS catalyzed reactions delphinidin, pelargonidin and cyanidin are reduced by an anthocyanidin reductase (ANR) to produce epi-flavan-3-ols (e.g. epicatechin). LAR and ANR are two exclusive enzymes in the biosynthesis of PA. The rest of the pathway is shared with anthocyanin. The monomers of PA, catechin and epicatechin, can induce oxidation in the cytosol and generate ROS, which is lethal for cells. Therefore, glucose or ascorbate conjugates of these flavan-3-ols are formed in the cytosol and get transported to the vacuole (Zhao and Dixon 2009; Yu et al. 2022a).

Recently, Jun et al. (2018) discovered species-specific diversity in PA composition. PAs in the leaves of *M. truncatula* are composed of only (−)-epicatechins. They discovered an interconversion between (+)-catechin to (−)-epicatechin catalyzed by an ANS homologue, leucoanthocyanidin dioxygenase (LDOX) (Fig. 2). LODX was first identified by analysing Arabidopsis *tds* mutants (*tds-4*) (Abrahams et al. 2003). Perhaps this may not be the case for pinto beans. In pinto bean seed coat tissues, catechin levels were found at higher levels in the CDC Pintium (RD) compared to 1533-15 (SD) (Duwadi et al. 2018). Moreover, Chen et al. (2015a) identified soluble flavan-3-ol monomers, soluble proanthocyanidin oligomers mostly with the B-type linkage and flavonoids (kaempferol, catechin and epicatechin) in the cranberry beans, which share similar phenotype as the pinto beans. They found a substantially higher amount of total phenolics in the regular darkening cranberry bean cultivars than the non-darkening ones (Chen et al. 2015a, b). Similarly, procyanidins were found abundantly in the coloured faba bean and lentils where their levels were genotype-specific (Elessawy et al. 2020).

### Transport and accumulation of PAs in the seed coat

Biosynthesis of PA monomers occurs at the cytoplasmic side of the endoplasmic reticulum (ER), where they get glycosylated, followed by their transport into the vacuole for polymerization as well as accumulation (Debeaujon et al. 2001; Zhao and Dixon 2009). An uridine diphosphate glucosyltransferase (UGT) catalyzes the formation of epicatechin-3′-*O*-glucoside in the cytosol by transferring the glucose molecule from UDP-glucose (Zhao et al. 2010). The transport mechanism is well studied for Arabidopsis, where PA polymers are exclusively composed of (−)-epicatechin. The epicatechin-3′-*O*-glucosides are transported into the vacuole through a multidrug and toxic extrusion (MATE) transporter, TT12 (Debeaujon et al. 2001; Marinova et al. 2007). In common bean, PvMATE8, an orthologue of Arabidopsis TT12 and *M. truncatula* MATE1, actively transports the epicatechin-3′-*O*-glucoside into the vacuole (Islam et al. 2022). Yu et al. (2022b) hypothesized an alternative pre-vacuolar polymerization of PAs in legumes. However, the LC-MS/MS analysis performed by Islam et al. (2022) found no PAs in the *tt12* Arabidopsis seeds. Instead, the *tt12* accumulated an abundance of epicatechin-3′-*O*-glucosides. Upon restoration of the transport function by the overexpression of *Pv*MATE8 in Arabidopsis *tt12* seeds, PA polymers (PA-A and PA-B) were formed, confirming the polymerization occurs in the vacuole. The monomer (+)-catechin is reported in the legume *M. truncatula*, where the transport mechanism is still under investigation (Yu et al. 2022a). A genome-wide analysis identified 59 MATEs in common beans and grouped them with MATEs from other plants with known functions (Islam et al. 2022). The cluster of MATEs that transport flavonoids are mostly TT12/ PA transporters, and other flavonol conjugates. Two other mode of transport of the flavonoid compounds include vesicle and glutathione-S-transferase (GST)-transport. Evidence of vesicle trafficking was reported by Ichino et al. (2020) where mutation in TT9, green fluorescent seed 9 protein, affect seed coat pigmentation in Arabidopsis. Furthermore, GST-like protein TT19, is partly involved for PA transport in Arabidopsis, as the seed coat endothelial tissues of *tt19* mutants contain PAs but the PA accumulation pattern was different (Debeaujon et al. 2001; Kitamura et al. 2004, 2010). Besides, an endomembrane proton pump, H^+^-ATPase (TT13/AHA10 in Arabidopsis), is also required along side of MATE for PA transport into the vacuole (Baxter et al. 2005; Appelhagen et al. 2015).

The PA polymers in the vacuole are colorless. They get transported to the apoplastic space of the cell wall where they turn brown in color. The mechanism by which PAs are transported and what signals the transport are not known yet. In the apoplast, the PAs react with several environmental oxidizing agents such as atmospheric O_2_, nitrogen oxides, UV lights, and enzymes polyphenol oxidase (PPO) and peroxidases (POD) (Pourcel et al. 2005, 2007). Oxidation converts colorless PAs (*ortho*-diphenols) to corresponding semiquinones and quinones, that ultimately react with other compounds to produce brown pigments on the seed coat. The PPO in Arabidopsis (also known as TT10) is a laccase like oxidase and upon reacting with the aromatic PA polymer, makes changes in the structure such that it appears brown. The PPO oxidation assay adopted for wheat did not detect any difference in PPO activity between RD and SD pinto bean lines (Marles et al. 2008). A recent study in peas (*P. sativum*) identified the association of PPO in the hilum coloration but not the seed coat (Balarynová et al. 2022). In pea cultivars, both the polymerization and oxidation of the predominant flavan-3-ol, gallocatechin are influenced by PPO (Ferraro et al. 2014).

### Postharvest seed coat darkening in pinto beans

Gesto and Vazquez (1976) first reported the possibility of an increase in phenol and phytate levels with seed coat darkening. Later, in both freshly harvested and aged seeds, Beninger et al. (2005) discovered that CDC Pintium (RD cultivar) had significantly higher levels of PAs than 1533-15 (SD cultivar). Recently, RD, SD, and ND lines showed differential levels of PA and its intermediate pathway metabolites in two separate studies in cranberry beans and pinto beans. Freixas Coutin et al. (2017) showed that the ND recombinant inbred lines (RILs) of cranberry beans do not accumulate PA in any of the early, intermediate, or mature seed stages, while the regular darkening beans produce a considerable amount of PA in all three stages. Duwadi et al. (2018) identified PA pathway intermediates catechin, cinnamic acid, and naringenin and flavonol derivatives, quercetin and kaempferol, at a significantly higher level in pinto bean cultivars CDC Pintium (RD) compared to 1533-15 (SD).

All commercial pinto beans available until recently were RD types. The newly developed SD pinto bean cultivars offer brighter colored seeds and hold their fresh look for a longer time (Elsadr et al. 2011). The ND beans are almost white and are not affected by the darkening effect. Although no particular nutritional difference was observed between freshly harvested and darkened pinto beans (Plhak et al. 1989), an association of faster cooking time and improved iron bioavailability with SD pintos (compared to RD) was observed by Wiesinger et al. (2021). They also found that these beneficial attributes in SD pinto beans compared to RD are due to lower levels of PAs.

Postharvest seed coat darkening in pinto beans can be reduced to some extent by storing them in cold, dry, and dark conditions and maintaining an inert atmosphere with a slightly higher nitrogen level (Barron et al. 1999). However, maintaining such conditions for a long period of time is expensive. Pinto beans are one of the staple foods in northern Mexico, which is also the home of the first commercially released SD cultivar, Pinto Saltillo (Sanchez-Valdez et al. 2004). A number of other commercial SD lines, such as 1533-15 [Canadian Food Inspection Agency (CFIA) Registration no. 6606], Slow Darkening Idaho Pinto-1 (SDIP-1), Kimberly, Shoshone, and ND-Palomino (Singh et al. 2008; Osorno et al. 2018) were developed later. Wit-rood boonje, a cranberry-like bean, carries the genetic component for the ND phenotype (Erfatpour et al. 2018). SD cultivars are the cultivar of choice for both consumers and bean growers due to their higher color stability over RD lines (Junk-Knievel et al. 2008). However, these newly developed SD pintos do not always show optimum agronomic performance when grown outside their adapted territories (Miklas et al. 2020).

### Genetics of postharvest seed coat darkening

**Fig. 3:**
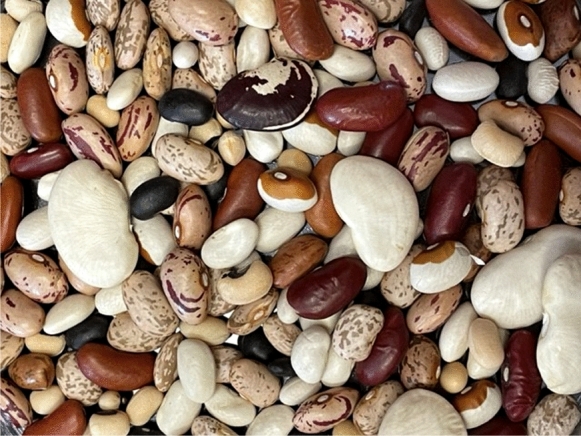
A photograph showing seed coat color of some popular market classes of common beans with wide color diversity, along with variation in their shapes, sizes and patterns

**Fig. 4:**
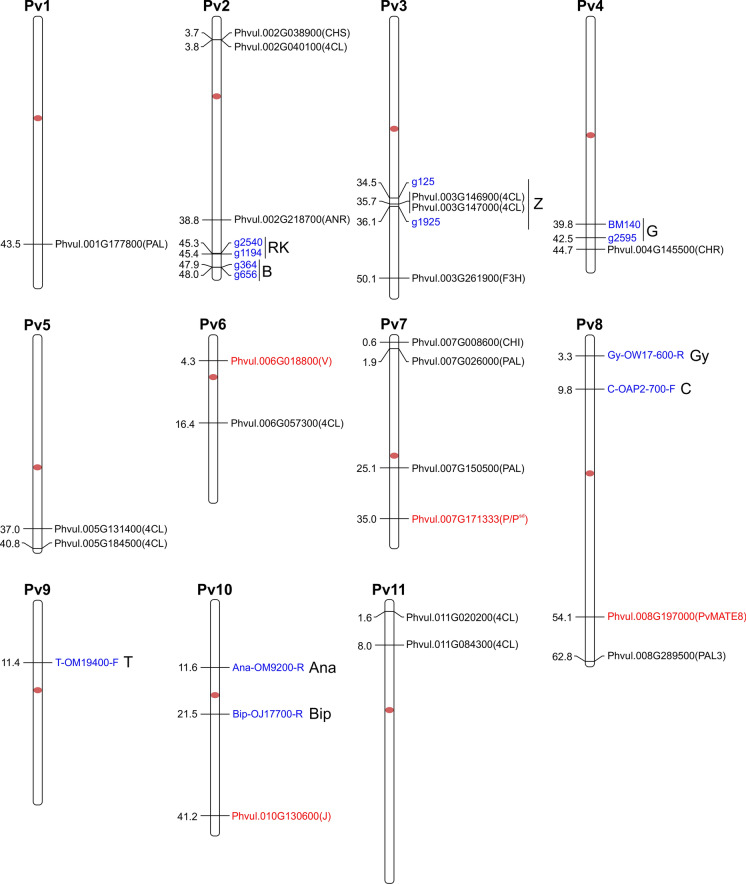
Chromosomal locations of color genes in common bean. Genomic distribution of putative biosynthetic genes (black), color and pattern genes (black) aligned with their closest markers (blue) and functionally characterized color genes (red) on *P. vulgaris* chromosome (Phytozome 13 https://phytozome-next.jgi.doe.gov/, accessed September 2023). The chromosome numbers are indicated above each chromosome and drawn to a scale in megabase pairs (Mbp). The chromosome size is indicated by its relative length using the information from Phytozome 13. Chromosome map illustrated by MapChart 2.32

Genetic resources are fundamental for crop improvement. Since the seed color, shape and size of dry beans is one of the key factors that influence consumer choice (Fig. 3), this qualitative trait has always been emphasized in bean breeding. Over the years, a handful of genes have been discovered, along with their several allelic forms, that are responsible for different seed coat colors and patterns. Genes at the loci *P*, *C*, *R*, *J/L*, *D*/*Z*, *G*, *B*, *V*, and *Rk* for seed coat base color and *T, Z, Bip*, and *Ana* for colored patterns have been reported in several common bean market classes (McClean et al. 2002). Among these genes, some are also involved in flower colors, indicating gene functions are developmental stage-specific. These genes follow either the dominance, epistasis, multi-, or co-dominance rules of genetics to produce different seed phenotypes (Bassett 2007). From the nine color coding loci only, *P*, *J*, and *V* are identified in the common bean genome by molecular characterization (Erfatpour and Pauls 2020; Islam et al. 2020; McClean et al. 2022). Among them, *P* (*P*^*sd*^ allele) and *J* control PA levels and postharvest seed coat darkening, while *V* determines flower color. The genomic investigation of potential phenylpropanoid pathway genes in *P. vulgaris* cultivar G18933 was carried out by Reinprecht et al. (2013). They also produced a chromosomal map that showed the locations of the color genes, their known markers, and the PA biosynthesis genes. Since then, tremendous progress has been made in the identification of common bean genes and more genome sequencing. The physical locations of color genes including the recently characterized ones and additional 4CL genes in common bean are indicated on an updated chromosomal map in Fig. 4.

The *Pigment (P)* gene is called the “ground factor” for determining the seed coat and flower color and patterns in beans, first described by Emerson (1909). Later, during the search for the gene determining the SD trait in pinto beans, Islam et al. (2020) identified the *P*^*sd*^ allele of P. P is a basic helix loop helix (bHLH) transcription factor that regulates *DFR* and *ANR* expression and PA biosynthesis. A recessive *P* allele (*pp*) produces white seeds and flowers (McClean et al. 2018). To date, multiple alleles of *P* have been discovered in common beans that have direct impact on seed coat color. For example, *p*^*gri*^ (*griseoalbus* character) for grey white flower and seed coat (Bassett 1994), *p*^*mic*^ (micropyle stripe) for seed coat with white micropyle stripe (Bassett and Miklas 2007), *p*^*stp*^ (stripped) and *p*^*hbw*^ (half banner white) for seed coat patterns (Bassett 1996). Furthermore, *P* also affects seed size and weight (McClean et al. 2018).

In addition to *P*^*sd*^, *J* is also associated with postharvest seed coat darkening (Bassett et al. 2002). *J* can function independently and/or pose an epistatic effect on other color genes. Recessive *J* alleles (*jj)* result in the ND phenotype (Elsadr et al. 2011). *J* is also responsible for seed coat patterns and shininess (Prakken 1970). When *J* is a major seed coat color gene, its allelic variant, *j*^*ers*^, controls a partly colored seed coat [reviewed in Bassett (2007)]. Pinto or cranberry beans with *jj* genotypes do not show postharvest darkening while recessive alleles of *P*^*sd*^ cause the SD phenotype (Elsadr et al. 2011; Islam et al. 2020). Furthermore, segregation analysis indicates that the *J* locus is epistatic to *P*^*sd*^; *J* determines the brown phenotype of the seed coat, while *P*^*sd*^ determines the rate or extent of darkening during postharvest storage.

### Regulation of PA biosynthesis

**Fig. 5:**
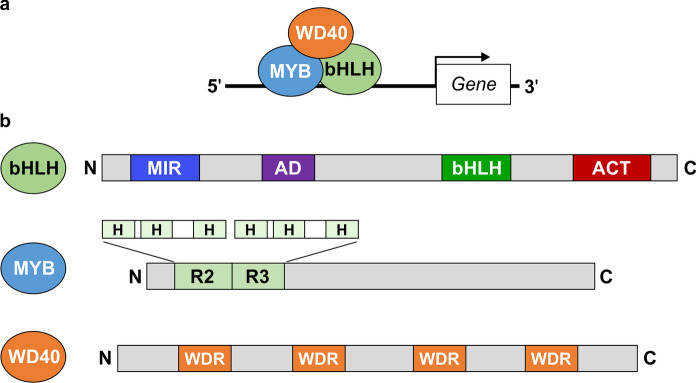
MBW complex proteins and PA gene regulation. **a** A schematic diagram showing MYB-bHLH-WD40 (MBW) complex binding to the target gene promoter for transcription activation. The upstream DNA region from translation start site of the gene is indicated by black line. **b** Protein motifs in bHLH, MYB and WD40. bHLH protein with MYB interaction region (MIR), Activation domain (AD), bHLH and ACT domain are shown, MYB protein with R2 and R3 motifs indicating the α-helices (H) involved in bHLH interaction and WD40 repeat (WDR) regions in WD40 proteins. Structures are not drawn to scale

In the phenylpropanoid biosynthetic pathway, genes encoding CHS, CHI, F3H, F3′H, and FLS are considered to be early biosynthetic genes (EBGs) that lead to the production of flavonoids, whereas other genes that encode enzymes DFR, ANS, ANR, LAR that lead to the production of PAs and anthocyanins are considered as late biosynthetic genes (LBGs) (Fig. 2). LBGs include both structural and transporter genes (Lepiniec et al. 2006). The regulatory mechanisms of these EBGs and LBGs vary in monocots and dicots (Petroni and Tonelli 2011). In monocots, both EBGs and LBGs are regulated by a ternary protein complex of a myeloblastosis (MYB) proto-oncogene homologue, a bHLH, and WD40 repeat proteins known as the MBW complex (Fig. 5a). While in dicots, MYB alone regulates EBGs, LBGs are regulated by the MBW complex (Gonzalez et al. 2008; Petroni and Tonelli 2011). Members of MBW complex proteins are encoded by multigene families and are the key to flavonoid biosynthesis (Ramsay and Glover 2005).

#### bHLH

The bHLH is a key regulatory member of the MBW complex. bHLH transcription factors are classified into groups and sub-groups based on their structural and functional properties (Heim et al. 2003). Eukaryotic bHLH proteins are classified into 26 subfamilies, where the subfamily IIIf is involved in anthocyanin and PA biosynthesis (Heim et al. 2003; Toledo-Ortiz et al. 2003). Subfamily IIIf bHLH contains an N-terminal MYB interaction region (MIR), followed by a poly-glutamate-containing activation domain repeat region, a bHLH region, and an ACT-like domain on the C-terminus (Fig. 5b) (Grotewold et al. 2000; Zimmermann et al. 2004). The MIR region of the bHLH protein interacts with R3 domain of the R2R3-MYB in the MBW complex. The function of the activation domain is not known, however, it is speculated to be involved in the interaction with the WD40 proteins (Heim et al. 2003). The 10–15 basic amino acids in the bHLH domain are crucial for recognizing a consensus E-box, CANNTG or G-box, CACGTG in the target promoter. The HLH region is formed with about 40 amino acids that are responsible for homo- and heterodimer formation (Heim et al. 2003; Pires and Dolan 2010). However, the C-terminal ACT domain is also found to be involved in homo- or heterodimer formation (Feller et al. 2006; Kong et al. 2012). In maize, the presence or absence of an ACT domain in the bHLH determines the regulation of two different gene targets. Lc, the first identified bHLH, interacts with the C1 (R2R3-MYB) to regulate anthocyanin production in maize (Ludwig et al. 1989). *Lc* is one of the loci of the *R1/B1* genes and regulates the flavonoid pathway genes in a tissue-specific manner (Ludwig and Wessler 1990). In Arabidopsis, GL3 and EGL3 are the bHLH regulators of anthocyanin biosynthesis. GL3 and EGL3 have overlapping and somewhat preferential functions in seedlings and flowers (Gonzalez et al. 2008). TT8 is the bHLH protein that regulates PA biosynthesis in Arabidopsis seeds (Nesi et al. 2000). All bHLH of subgroup IIIf maintain a feedback loop by regulating themselves and their target structural genes by interacting with the MBW complex (Baudry et al. 2004; Albert et al. 2014; Xu et al. 2015). bHLH can also interact with MYB repressors and interfere with MBW complex function (Jun et al. 2015).

In common beans, the major seed coat color determinant gene *P* encodes a bHLH (Emerson 1909; McClean et al. 2018). *P* is a homolog of Mendel’s famous *A* gene in pea. Islam et al. (2020) identified that an allele of the *P* gene, *P*^*sd*^, is responsible for the SD trait in pinto beans. An InDel and a SNP in the AD and bHLH motifs (Fig. 5b) resulted in reduced activity of the *P*^*sd*^ allele. Homozygous alleles of *P*^*sd*^ resulted in the decreased expression of *DFR* and *ANR*, thereby reducing the PA levels.

#### MYB

The MYB proteins belong to one of the largest transcription factor families and are major regulators of the PA biosynthetic genes (Dubos et al. 2010). MYBs exist in eukaryotes with multifunctionality and wide diversity (Jin and Martin 1999). They are classified into different subfamilies depending on the presence of imperfect adjacent repeats (R1, R2, R3, and R4) in the MYB DNA binding motifs (Rosinski and Atchley 1998). Each of the repeats is about 52 amino acids, forming three α-helices (Dubos et al. 2010). A Helix-Turn-Helix (HTH) is formed between the 2nd and 3rd helices, and the 3rd helix makes contact with the DNA. The most abundant type of MYBs in plants is R2R3-MYBs, which contain two adjacent repeats (Fig. 5b). R2R3-MYB members regulate PA and anthocyanin biosynthesis (Dias et al. 2003; Ramsay and Glover 2005; Pireyre and Burow 2015). MYBs recognise either the MYB core elements (5′-CNGTTR-3′, also called Myb Binding Sites/MBS) or “AC-rich” elements (5′-[A/C]CC[A/T]A[A/C]-3′) on the target promoter. The COLORLESS 1 (C1) is the first identified plant R2R3-MYB which regulates *A1* gene expression in maize kernels (Paz-Ares et al. 1987). A paralogue of C1, Pl, was identified later that regulates the same gene but in the vegetative and floral tissues of maize (Cocciolone and Cone 1993). In Arabidopsis, functionally redundant R2R3-MYBs (MYB11, MYB12 and MYB111) are identified in all tissues that regulate the EBGs without forming a MBW complex (Stracke et al. 2007). The R2R3-MYB activators of LBGs for the production of anthocyanin in flowers and plant bodies are PAP1, PAP2, MYB113, MYB114, whereas TT2 is involved in the regulation of PAs in the seed coat (Nesi et al. 2001; Gonzalez et al. 2008). Nevertheless, multiple MYBs are often found to co-regulate a single gene transcription (Schaart et al. 2013; Albert et al. 2014). MYBs can function as both activators and repressors. Among numerous MYBs involved in PA biosynthesis, repressors are also present. For example, in Arabidopsis, two R3-MYBs, MYBL2 and CPC, interfere in the MBW complex formation thus negatively regulating anthocyanin and PA biosynthesis (Dubos et al. 2008; Zhu et al. 2009). Similarly, MYB2 (R3R3-MYB) suppresses MBW complex function in *M. truncatula* (Jun et al. 2015).

Erfatpour and Pauls (2020) identified that the ND trait is controlled by a MYB protein, J. Recessive alleles of *J* is responsible for the ND phenotype (Freixas Coutin et al. 2017). It is very likely that, J regulates PA biosynthesis, and its loss of function causes the complete elimination of PAs in the seed coats of cranberry and pinto beans. However, it is not known if J is a member of the MBW complex for the regulation of PA biosynthesis or if it regulates PA levels *via* an alternate route.

#### WD40

The WD40 protein in the MBW complex provides a scaffold for holding the concomitant MYB and bHLH for the intended function. The constitutively expressed WD40 repeat protein family is characterized by the presence of a 40 residue core region of a glycine-histidine (GH) and a tryptophan-aspartate (WD) dipeptide (Fig. 5b) (Ramsay and Glover 2005). The first WD40 protein-coding locus, *AN11*, was identified in petunia for anthocyanin biosynthesis, followed by *PAC1* in maize (De Vetten et al. 1997; Carey et al. 2004). Later, interaction studies in an MBW complex led to the discovery of Arabidopsis TTG1 (WD40) (Walker et al. 1999; Zhang et al. 2003; Zhang and Schrader 2017). TTG1 controls a variety of cellular fates including PA and anthocyanin biosynthesis, root hair pattern, trichome and seed mucilage formations. For each of these functions, a specific bHLH and a MYB partner are recruited, that determine the tissue- and developmental stage-specific roles, respectively (Koornneef 1981; Debeaujon et al. 2003). No WD40 has yet been characterized in common bean.

#### Other regulatory factors in the in the MBW complex

The core MYB, bHLH, and WD40 proteins form the primary skeleton of the MBW protein complex, while additional factors have also been identified that either stabilize or destabilize the complex (Lloyd et al. 2017). One such interactor protein of the MBW complex (TT2-TT8-TTG1) in Arabidopsis is TTG2, a WRKY transcription factor (Gonzalez et al. 2016). The TT2-TT8-TTG1-TTG2 complex regulates the expression of *TT12* (encodes the MATE transporter). It was hypothesized by Lloyd et al. (2017) that the MBW complex either transcriptionally activates *TTG2* or forms a complex to regulate *TT12* expression and thus control the PA accumulation in seeds. Additionally, repressor mediated inactivation and post-translational modifications of the MBW complex proteins can affect seed coat darkening in common bean. For instance, several R3 MYBs were reported to repress the MBW complex function in Arabidopsis (Dubos et al. 2008; Zhu et al. 2009), M. *truncatula* (Jun et al. 2015) and petunia (Albert et al. 2014). MicroRNA-targeted SQUAMOSA PROMOTER BINDING PROTEIN-LIKE (SPL) 9 can play a dual role as a transcription activator or suppressor. A high level of SPL9 represses *DFR* expression. SPL9 dissociates the TT8 and TTG1 from the MBW complex post-translationally and upon stress signals by binding with PAP1 in anthocyanin biosynthesis (Gou et al. 2011). Moreover, it is hypothesized that the mechanism of dissociation by SPL9 is required for prioritizing PAP1 to TT2 in the branching of anthocyanin to PA biosynthesis, respectively, based on spatial and temporal signals. Similarly, miR858b blocks the activity of DkMYB19 and DkMYB20 in persimmon fruits, thus reducing the PA levels (Yang et al. 2020). Recently, Yang et al. (2022) summarized the role of several micro-RNAs and SPLs on flavonoid pathway which mostly include anthocyanin biosynthesis but much is known for PA biosynthesis.

## Concluding remarks

Diverse growth habitats, high nutritional value, an affordable price, and long storage time have made dry beans the most popular legume worldwide; however, postharvest seed coat darkening poses a significant problem. Two regulatory genes of the PA biosynthetic pathway, *P*^*sd*^ and *J*, have been identified in common bean, that control SD and ND phenotypes, respectively. It has been established through molecular characterization that P (or P^sd^) controls the LBGs of the PA pathway. P orthologs of bHLH proteins require protein partners in other plant species for their complete function. The interacting partners of P in common beans are still unknown. It is not yet known if J (a MYB protein) is P’s MYB partner in the common bean MBW complex or if another MYB fulfills this function. In a similar manner, it is necessary to identify the WD40 and other plant factors (proteins, microRNA, siRNA, etc.,) that are involved in the complex with P. The unidentified component(s) could function either as a bridge between the proteins (e.g. connecting WD40 with other components) or dissociate any suppressors of the interaction. The unavailability of mutant lines for WD40 in beans makes it challenging for elucidating the overall PA regulatory network. However, the discovery of the *Pv*MATE8 transporter clarifies the manner in which PA accumulates in the vacuoles.

Seed coat color is a complex trait that is associated with PA levels. A complete understanding of the PA biosynthetic genes and their regulation is extremely important to develop pinto beans with desired seed coat color. Recent findings on the color of bean seed coats has created new milestones for improving variety development and preventing millions of dollars in market losses.
